# Synergistic Anti-Cancer Effects of Curcumin and Thymoquinone Against Melanoma

**DOI:** 10.3390/antiox13121573

**Published:** 2024-12-20

**Authors:** Hana Mohd, Bozena Michniak-Kohn

**Affiliations:** 1 Ernest Mario School of Pharmacy, Rutgers-The State University of New Jersey, 160 Frelinghuysen Road, Piscataway, NJ 08854, USA; hm527@dls.rutgers.edu; 2 Center for Dermal Research, Rutgers-The State University of New Jersey, 145 Bevier Road, Piscataway, NJ 08854, USA

**Keywords:** curcumin, thymoquinone, melanoma, RNAseq, cancer

## Abstract

Combining anti-cancer agents in cancer therapies is becoming increasingly common because of their improved efficacy, reduced toxicity, and decreased risk of resistance development. Melanoma, a highly aggressive form of skin cancer characterized by limited treatment options due to chemoresistance, poses a considerable challenge for effective management. Here, we test the hypothesis that dietary supplements such as thymoquinone (TQ) and curcumin (CU) cooperatively modulate cancer-associated cellular mechanisms to inhibit melanoma progression. Through a series of in vitro experiments utilizing the A375 melanoma cell line, including assessments of cell viability, apoptosis, multicellular tumor spheroid models, reactive oxygen species (ROS) quantification, metabolomics analysis, and RNA sequencing, we established that the combined application of TQ and CU exhibited superior anti-tumor effects compared to their individual use. Our results indicate that the combination treatment significantly inhibited cell viability and induced apoptosis more effectively than either agent alone, with optimal synergy observed at concentrations of 25 µM CU and 10 µM TQ against A375 cells. Additionally, the combination treatment markedly elevated ROS levels, selectively activating the mitochondrial apoptotic pathway via caspase-9. Differential gene expression analysis further revealed a unique synergistic effect of the combination treatment, with enhanced regulation of genes related to oxidative stress and apoptosis. Notably, pathways such as mitochondrial apoptotic signaling and redox homeostasis were more effectively influenced by the combination, with genes such as GPX3, CYP4F11, and HSPB8 cooperatively regulated. Overall, the findings suggest that, in combination, TQ and CU acts synergistically against melanoma; however, further experimental and clinical studies are required to confirm its therapeutic potential.

## 1. Introduction 

Melanoma represents around 1.7% of all newly diagnosed primary malignant cancers worldwide and accounts for nearly 0.6% of total cancer mortality [1]. As one of the most severe forms of skin cancer, melanoma originates in melanocytes, the cells responsible for producing the skin pigment melanin [2]. Although the exact causes of melanoma are not fully understood, UV radiation exposure is a significant risk factor [2]. Melanoma is more prevalent among people under 40, particularly women [1]. While it constitutes about 1% of all malignant skin tumors, cutaneous malignant melanoma is considered the deadliest and most aggressive skin cancer phenotype [3].

A significant challenge in treating and managing cancers like melanoma is the development of chemoresistance. Cancer cells often develop compensatory mutations or activate substitute signaling pathways in response to chemotherapy, leading to relapse [4]. Changing to a different agent after relapse is usually ineffective because of resistance to the new treatment. In contrast, combining two or more phytochemicals can provide significant advantages over monotherapy by targeting multiple pathways simultaneously, reducing the likelihood of resistance [5]. This approach can also decrease toxicity, enhance efficacy, and produce a synergistic effect, whereby the combined therapeutic impact exceeds the sum of individual effects [6,7]. Different phytochemicals may act on various molecular targets or signaling pathways in cancer progression, providing a comprehensive treatment strategy [8].

Additionally, combining agents can achieve desired therapeutic outcomes with lower doses, potentially reducing adverse side effects [9]. Some agents may complement each other by increasing their effectiveness, thus inhibiting tumor growth, proliferation, and metastasis more effectively than a single agent alone. Recent findings suggest that various dietary compounds exhibit anti-tumor properties by influencing several oncogenic pathways. Researchers now believe that alternative medicine holds promising sources for new anti-cancer treatments [10]. In recent decades, interest in medicinal herbs and plants has grown due to their minimal complications and fewer side effects than conventional chemotherapy.

Moreover, the World Health Organization has encouraged developing countries to incorporate traditional medicinal plants into their primary healthcare programs [11]. Two dietary agents, curcumin (CU) and thymoquinone (TQ), have been widely studied for their anti-cancer properties in melanoma. CU, a polyphenol extracted from turmeric, demonstrates chemo-preventive and anti-tumor effects by regulating transcription factors and signaling pathways, such as the PI3K/mTOR, GSK-3beta, and Ras/Raf/MEK pathways [5,12,13]. TQ, the active component of nigella sativa seeds, has shown antioxidant and anti-inflammatory effectiveness in models of asthma, encephalomyelitis, neurodegeneration, diabetes, and cancer development [14]. Studies have shown that TQ exerts its anti-neoplastic effects by inhibiting cell proliferation, inducing apoptosis, arresting the cell cycle, generating reactive oxygen species (ROS), and inhibiting metastasis and angiogenesis [15]. TQ also suppressed caspase-1 activity, reduced IL-1β and IL-18 secretion, and hindered melanoma cell migration by targeting the NLRP3 subunit of inflammasomes [16]. Considering their diverse anti-cancer mechanisms, we hypothesized that co-administering TQ and CU could synergistically modulate multiple cancer-related signaling pathways, thereby enhancing effectiveness against melanoma. In this study, through systematic assays and bioinformatics analyses, we report for the first time that the combination of CU and TQ exhibits a superior anti-tumorigenic effect in skin cancer.

## 2. Materials and Methods 

### 2.1. Cell Culture

The A375 human melanoma cell lines, primary human dermal fibroblast adult (HDFa) cell lines, and HaCaT human keratinocytes cell lines were purchased from the ATCC, VA, USA, and AddexBio, San Diego, CA, USA. The cells were cultured in Dulbecco’s Modified Eagle Medium (DMEM), complemented with 10% fetal bovine serum (FBS) and 1% penicillin/streptomycin. Cells were cultured under conditions of 5% CO_2_ at 37 °C. When required, the cells were rinsed with PBS and detached using a trypsin solution (0.025% trypsin and 0.02% EDTA; Sigma-Aldrich, St. Louis, MO, USA).

### 2.2. Alamar Blue Viability Assay

The cell viability was assessed using the Alamar Blue assay, which evaluated cytotoxicity induced by various agents. This assay was used to determine the impact of TQ (Sigma-Aldrich) and CU (Sigma-Aldrich) on the viability of A375, HDFa, and HaCaT cells. Cells were seeded onto 96-well plates at a density of 1 × 10^4^ cells per well and incubated overnight at 37°C in 5% CO_2_. The cells were incubated overnight to allow for adhesion, followed by treatment with varying concentrations of TQ and CU and their combination for 24 h. The fluorescence intensity was measured using a microplate reader (Tecan, Männedorf, Switzerland) at excitation and emission wavelengths of 540 nm and 595 nm, respectively. The results were averaged over three different independent experiments (*n* = 3) with three replicates per experiment.

### 2.3. Multicellular Tumor Spheroid Culture (MCTS) and Imaging

Human melanoma cells (A375) obtained from ATCC were grown in DMEM medium supplemented with 10% fetal bovine serum (FBS) and 0.1% penicillin–streptomycin and amphotericin-β. The A375 cells were cultured in a T-75 flask and incubated at 37 °C with 5% CO_2_. Once reaching 80% confluency, the cells were harvested from the T-75 flask and seeded at a density of 4000 cells per well in 200 μL of medium containing 10% FCS in a low attachment NunclonSphera 96-well ultra-low attachment plate (Thermo Scientific Nunc™, Waltham, MA, USA). Cells were incubated for 72–96 h until the multicellular tumor spheroids (MCTSs) reached an average diameter of 450 μm. 

To analyze the live/dead status in A375 MCTSs, dual-color fluorescent staining was conducted using Calcein-AM and propidium iodide (PI), both obtained from Sigma-Aldrich. Following spheroid formation, A375 spheroids were treated with 25 µM curcumin (CU), 10 µM thymoquinone (TQ), and their combination. After 24 h of treatment, MCTSs were incubated with 3 µM Calcein-AM and 5 µM PI solutions for 30 min in the dark at 37 °C and then directly imaged using an ECHO Revolve Microscope (Model RVL-100-M, BICO Company, San Diego, CA, USA, Serial #: M-00395-RVL) (20×/0.4 objective).

### 2.4. Viability Measurement in Spheroids

For measuring spheroid viability, CellTiter-Glo 3D (CTG3D; Promega, Madison, WI, USA) was used to determine ATP levels from spheroids, following the manufacturer’s instructions. CTG3D was added in a 1:1 volume ratio and incubated for 30 min (for cells) or 90 min (for spheroids) in each ULA well. The plates were shaken using Teleshake shakers (420 rpm for 2 min) and then incubated at room temperature for 10 to 30 min before measuring luminescence with a microplate reader (Tecan, Männedorf, Switzerland). The absolute luminescence values were normalized, with the luminescence signal from untreated cells or spheroids considered 100% viability. Relative viability was calculated as the percentage of luminescence signals from cells or spheroids treated versus untreated with drugs.

### 2.5. Apoptosis Assay

For Annexin V staining, cells were grown in 60 mm plates until reaching 90% confluence, treated with 25 µM curcumin, 10 µM thymoquinone (TQ), or their combination, then trypsinized and counted. According to the manufacturer’s instructions, cells were stained with Annexin V–FITC (BD Pharmingen, Franklin Lakes, NJ, USA). Cells were analyzed for FITC and PI fluorescent signals using FL1 and FL2 detectors of the flow cytometry (Beckman Coulter, Brea, CA, USA), respectively (λex/em 488/530 nm for FITC and λex/em 535/617 nm for PI).

### 2.6. Measurement of Intracellular ROS Generation

The effect of TQ and CU on inducing intracellular ROS in A375 cells was evaluated using flow cytometry (Beckman Coulter, Brea, CA, USA), using a method modified from a previously published study by Yang et al. [17]. Briefly, 2 × 10⁵ A375 cells were plated in 60 mm plates for 24 h and then treated with 10 µM TQ, 25 µM CU, or their combination for 24 h. The ROS levels were measured using CM-H2DCFDA (Invitrogen, Carlsbad, CA, USA). Cells were treated with 10 µM CM-H2DCFDA in cell culture medium without FBS at 37 °C for 40 min, followed by three washes with phosphate-buffered saline (PBS). The treated cells were collected, and their fluorescence intensity was promptly measured using a flow cytometer equipped with an FL-1 filter, with emission at 525 nm and excitation at 480 nm.

### 2.7. Caspase Activity Assay 

Caspase activities were assessed using the Caspase-Glo 8 and Caspase-Glo 9 Assays from Promega (Madison, WI, USA). The A375 cells were plated in a 96-well microtiter plate (Corning, Corning, NY, USA) at a concentration of 1 × 10⁴ cells per well. Then, the cells were exposed to 10uM of TQ, 25 μM of CU, and their combination for 24 h. Following the manufacturer’s instructions, 100 µL of the reagents were added after incubation, and the plates were kept in the dark at room temperature for 30 min. Luminescence was then measured using a microplate reader (Tecan, Männedorf, Switzerland).

### 2.8. LC-MS Metabolomic Analysis

Metabolomic analysis using LC-MS was performed at the Metabolomics Shared Resources, Rutgers Cancer Institute of New Jersey (CINJ), according to the protocol previously described by Li et al. [17,18]. The A375 cells were seeded at a density of 3 × 10⁶ cells per 10 cm culture plate (*n* = 3) and cultured overnight in DMEM supplemented with dialyzed FBS. The cells were treated for 24 h with 10 μM of TQ, 25 μM of CU, and their combination. After three PBS washes, the cells were scraped and centrifuged at 15,000× *g* for 10 min at 4 °C. Metabolites were extracted using 1 mL of a cold 40:40:20 methanol: acetonitrile: water containing 0.5% formic acid. The mixture was incubated on ice for 5 min and then neutralized with 50 µL of 15% NH4HCO3. The supernatant obtained was utilized for the LC-MS analysis.

The LC separation was performed using an XBridge BEH Amide column (2.1 mm × 150 mm, 2.5 µm particle size, 130 Å pore size; Waters, Milford, MA, USA). The mobile phase consisted of the following two solvents: Solvent A, a mixture of water and acetonitrile (95:5, *v*/*v*) containing 20 mM NH4AC and 20 mM NH4OH at pH 9, and Solvent B, a blend of acetonitrile and water (80:20, *v*/*v*) with 20 mM NH4AC and 20 mM NH4OH at pH 9. The flow rate was set to 300 µL/min, with an injection volume of 5 µL, and the column temperature was maintained at 25 °C. Mass spectrometry was performed in both positive and negative ion modes, using a resolution of 70,000 at *m*/*z* 200, an automatic gain control target of 3 × 10^6^, and a scan range of *m*/*z* 72–1000.

### 2.9. Isolation of Nucleic Acids and Next-Generation Sequencing (NGS)

A375 cells were plated in 10 cm culture dishes at a density of 2 × 10⁵ cells per dish and left to adhere overnight. The cells were then treated with 10 µM TQ, 25 µM CU, or a combination of both. Total RNA was isolated using the AllPrep RNA Mini Kit (Qiagen, Valencia, CA, USA). The integrity and concentration of the RNA were evaluated with an Agilent 2100 Bioanalyzer and a NanoDrop spectrophotometer. For RNA sequencing (RNAseq), 3 µg of RNA from each sample was utilized. Library preparation and sequencing were carried out by RUCDR Infinite Biologics. RNA libraries were prepared using the Illumina TruSeq RNA preparation kit (Illumina, San Diego, CA, USA) and sequenced on an Illumina NextSeq 500 platform, generating 75 bp single-end reads with a depth of 30–40 million reads per sample. Additional information on standard RNAseq methodologies can be found in the work of Gue et al. [19].

### 2.10. Differential Expression Analysis

A differential expression analysis was conducted using the DESeq2 package (v1.18; R v3.6.1). DESeq2 was used to compare gene expression among the groups of samples. The Wald test was applied to generate *p*-values and log2 fold changes. Genes with an adjusted *p*-value of less than 0.05 and an absolute log2 fold change greater than 1 were identified as differentially expressed genes.

### 2.11. Quantitative Real-Time Polymerase Chain Reaction (RT-PCR)

The mRNA was obtained using a kit from Thermo Fisher Scientific. First-strand cDNA was made from 1 µg of the extracted RNA using the SuperScript III First-Strand cDNA Synthesis System (Invitrogen, Grand Island, NY, USA). Real-time PCR was executed to analyze the mRNA expression of GPX3, CYP4F11, CASP-3, H1F1, and HSPB8 genes, with cDNA as the template and Power SYBR Green PCR Master Mix (Applied Biosystems, Carlsbad, CA, USA) as the reagent. Primers were procured from Integrated DNA Technologies (IDT, Coralville, IA, USA). mRNA expression levels were quantified as fold changes and normalized to GAPDH expression using the 2^−ΔΔCT^ method, with GAPDH serving as the internal control.

### 2.12. Statistical Analysis

The results are expressed as the mean ± SEM or SD, with a *p*-value ≤ 0.05 considered statistically significant. Statistical analysis was performed using a two-tailed unpaired Student’s *t*-test for comparisons between two groups and one-way ANOVA followed by Dunnett’s post hoc test for multiple group comparisons, utilizing GraphPad Prism. Metabolomic pathway and pathway enrichment analyses were conducted using the Web-based tool MetaboAnalystR 5.0 (https://www.metaboanalyst.ca (accessed on 18 November 2024)).

## 3. Results and Discussion 

### 3.1. Curcumin and Thymoquinone Synergistically Inhibited Cell Viability in A375 Melanoma Cancer Cells

**Figure 1:**
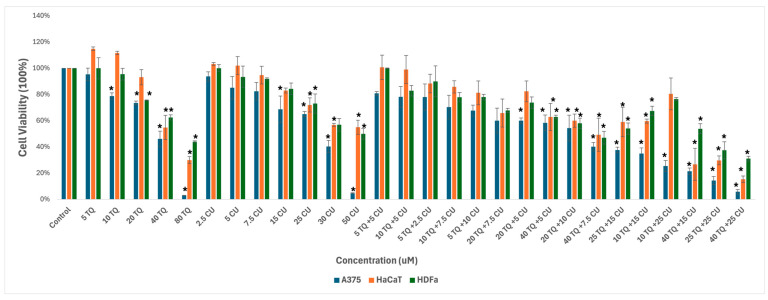
Effect of thymoquinone, curcumin, and their combination on the viability of human (A375) metastatic melanoma cells and the two healthy human keratinocyte (HaCaT) and human dermal fibroblast (HDfa) cell lines. The values are presented as the percentage of cell death. The data represent the mean ± SD of three independent experiments conducted in triplicate (vs. control, * *p* < 0.001).

We used melanoma and healthy human skin cell lines to study the effect of the combination of CU and TQ. A375, HDFa, and HaCaT were treated with serial dosages of CU (2.5 to 50 µM) and TQ (5 to 80 µM) individually and their combination for 24 h to determine growth inhibitory effects using the AlamarBlue assay (Figure 1). 

The individual treatments and their combination are safe for healthy skin cell lines at low concentrations, ranging from 5 µM to 20 µM for TQ and 2.5 µM to 25 µM for CU.

**Figure 2:**
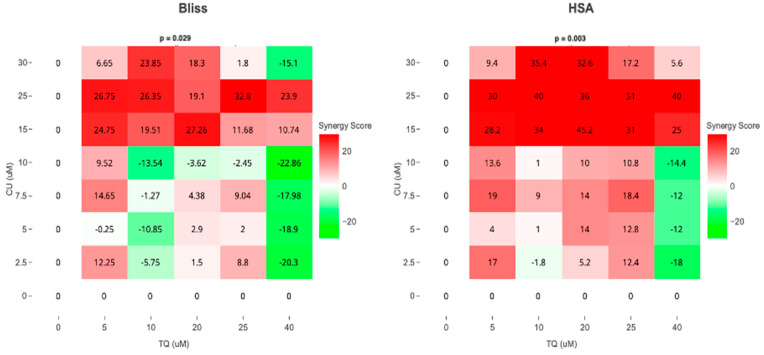
Synergism between TQ and CU in A375 cells. Bliss and HSA synergy scores (SynergyFinder.com) were calculated to predict the potential synergism of TQ and CU.

As a result, we move forward to test it on the melanoma cell line. Cell viability, as measured by the AlamarBlue assay, revealed that combined treatment with TQ and CU potentiated the growth inhibitory effect on A375 cell lines after 24 h of compound exposure. Individual treatment with TQ alone provided only marginal growth inhibition (5% to 26%) at low concentrations (5 to 20 µM). Still, it was substantially more effective at inhibition of growth (54%) with the highest concentration (40 µM). The CU-only treatment exhibited a dose-dependent response with a much more potent inhibition (6% to 95%) of growth at all concentrations (2.5 to 50 µM). We found that the combination was more effective at inhibiting cell proliferation than the individual agents but not across all combination ranges (Figure 1). As a result, we conducted the Combination Index (CI) calculation using SynergyFinder.com. To assess the potential cooperativity between TQ and CU, we calculated the CI for the TQ-CU combinations using two methods. The first method, the ‘Highest Single Agent’ (HAS) method (Figure 2), evaluates whether the combination effect is more significant than the individual agents. The second method employed the ‘Bliss Independence’ model (Figure 2), which calculates the multiplicative probability, assuming the agents act independently toward an expected outcome. The CI derived from both methods indicates strong cooperative interactions between CU and TQ, with an antagonistic effect observed at specific concentrations, such as 40 µM TQ combined with 2.5 µM CU. The highest synergism was observed at 25 µM CU and 10 µM TQ, which also resulted in cellular viabilities of 80% in HaCaT cells and 76% in HDFa cells. Therefore, we proceeded with these concentrations for further experiments.

**Figure 3:**
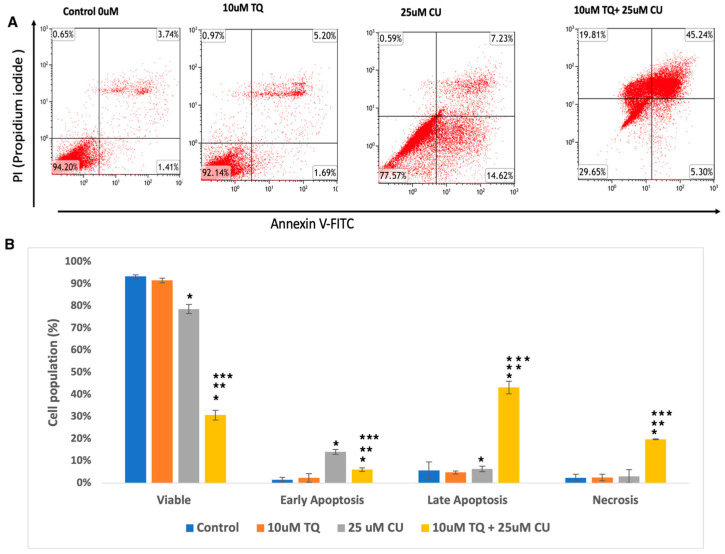
(**A**) Representative histograms obtained using flow cytometry on an apoptosis assay from various groups are shown (quadrant: upper left—dead cells; upper right—late apoptosis; lower left—live cells; lower right—early apoptosis); (**B**) percentage of viable, dead, early apoptotic, and late apoptotic cells in the control and compound-treated groups. *n* = 3; value are presented as the mean ± SD. * *p* < 0.05 vs. control cells; ** *p* < 0.05 combination vs. 25 μM CU; *** *p* < 0.05 combination vs. 10 μM TQ.

We evaluated whether the decrease in cellular growth observed in the cell proliferation assay by the TQ-CU combination was due to apoptosis using a flow cytometry-based assay and from the analysis determined the proportion of early and late apoptotic cells associated with each treatment. Both CU (25 μM) and TQ (10 μM) induced apoptosis in A375 cells, and their combination further significantly enhanced apoptosis rates (Figure 3A,B). Figure 3 displays the proportion of apoptotic and live cells within the entire population resulting from each treatment. The individual treatment concentrations included 25 µM CU, 10 µM TQ, and the combination treatment with 25 µM CU + 10 µM TQ. The combination treatments increased the number of late apoptotic cells by 6.25- and 8.7-fold compared to CU and TQ alone, whereas early apoptosis only increased by a small fold. These findings indicate that the combination significantly induced apoptosis compared to the control (Figure 3B), with notable rises in both early and late apoptotic cells, as determined by ANOVA (*p* < 0.01, *n* = 3). These results align with a study by El-Far et al., who found that the combination of TQ and CU significantly improved apoptosis in a breast cancer cell line compared to each drug alone [20]. 

Overall, these data indicate that the combination of TQ and CU exhibits superior anti-cancer effects compared to the individual agents. Notably, CU TQ, and their combination significantly induced apoptosis in A375 cells and inhibited their progression. Several studies have demonstrated the anti-cancer effects of either CU or TQ against A375 cell lines. [21,22,23,24,25]. However, no literature considers the combinatory benefits of CU and TQ against both on a melanoma cell line. 

### 3.2. Curcumin and Thymoquinone Synergistically Inhibited Cell Viability in A375 Melanoma Cancer Cells in the Spheroid Model 

In 2D model systems, cells are uniformly exposed to oxygen, nutrients, and other biochemical signals, whereas in 3D models, cells experience gradients of these conditions, resulting in cellular layers with different proliferative capacities and drug responses [26]. Multicellular tumor spheroids (MCTSs) are heterogeneous cell clusters that function as 3D models, closely replicating the characteristics of solid tumors in vitro [27].

**Figure 4:**
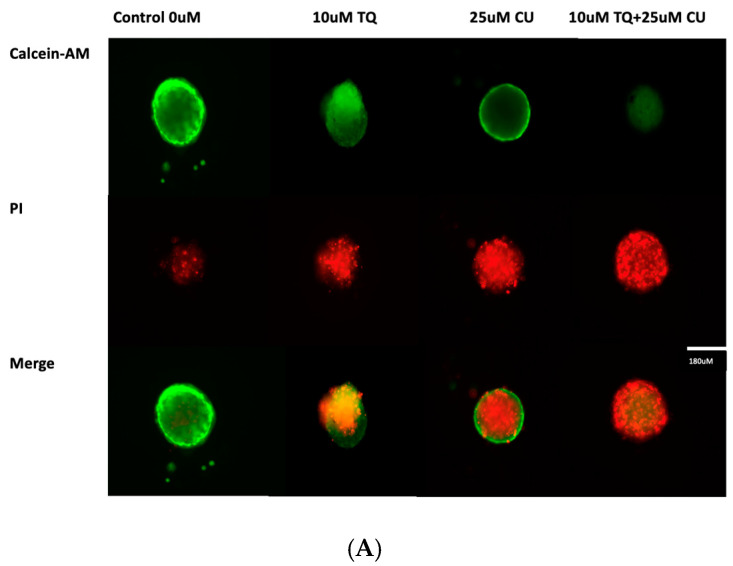

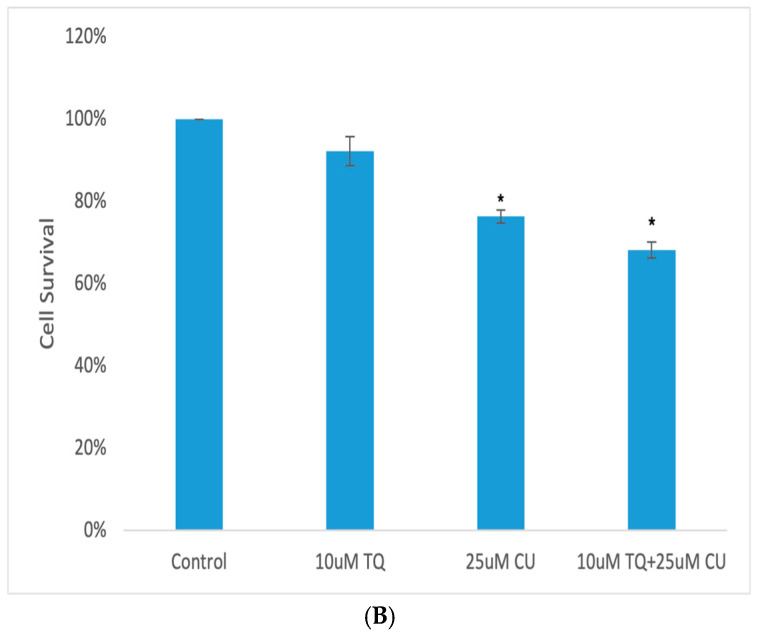
(**A**) Calcein-AM/PI dual-stained A375 multicellular tumor spheroids after 24 h of treatment with CU, TQ, or their combination. A red signal indicates dead cells, and a green signal indicates viable cells. Images obtained using an ECHO Revolve Microscope (Model RVL-100-M, Serial #: M-00395-RVL) (20×/0.4 objective). Scale bar: 180 µM. (**B**) Effect of thymoquinone, curcumin, and their combination on the viability of human (A375) metastatic melanoma cells in an MCTS model using a CellTiter-Glo 3D assay.*p*-Values are presented as the mean ± SD. * *p* < 0.05 vs. control.

A 3D model of A375 cells was initiated to investigate the efficacy of CU, TQ, and their combination on cell viability after 24 h of drug incubation. The data reveal that the A375 cells in MCTSs exhibited one-fold lower sensitivity to 25 µM CU, one-fold lower sensitivity to 10 µM TQ, and two-fold lower sensitivity to their combination compared to 2D cell cultures (Figure 4B). This reduced sensitivity is attributed to the spheroid structure, which impedes drug penetration, and the cellular heterogeneity within the spheroids [28].

A live/dead analysis of the A375 MCTSs was performed after 24 h of treatment with 25 µM CU, 10 µM TQ, and their combination, using dual-fluorescence staining with Calcein-AM and PI dyes (Figure 4A). Calcein-AM/PI dual staining demonstrated the maintained compactness of the control spheroids, which predominantly contained live, green, and fluorescent cells, with a small proportion of red-fluorescing cells observed in the core. This may be attributed to the overgrowth of the spheroid, leading to oxygen and nutrient deprivation, ultimately, resulting in cell death. [28,29]. Our data reveal that the combination of CU and TQ exhibited synergistic cytotoxicity against A375 cells in the 3D spheroid model and 2D.

### 3.3. Curcumin and Thymoquinone Induce Oxidative Stress in A375 Cells Using the Mitochondrial Pathway

**Figure 5:**
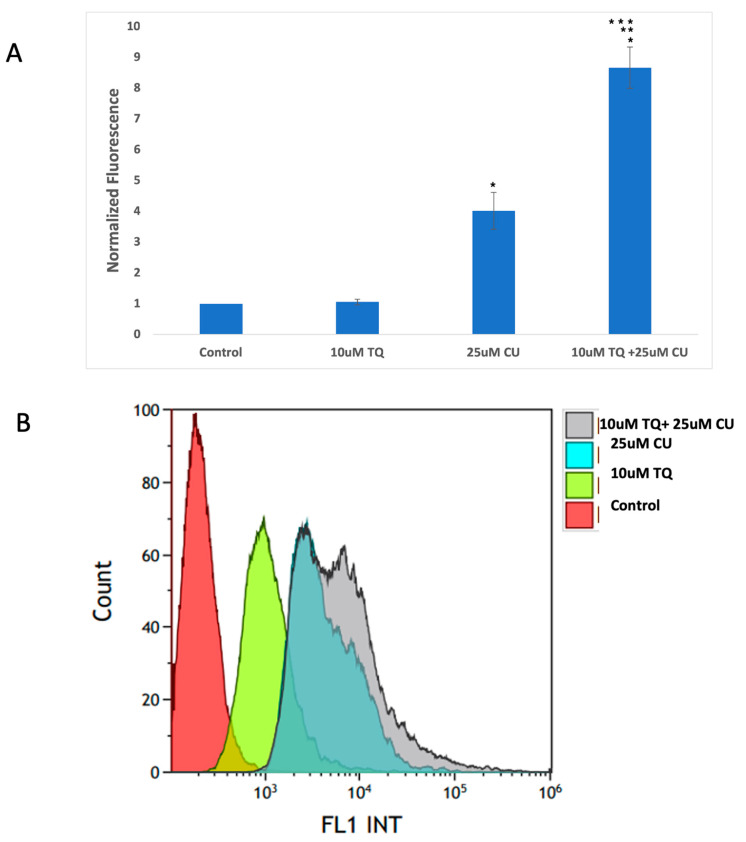

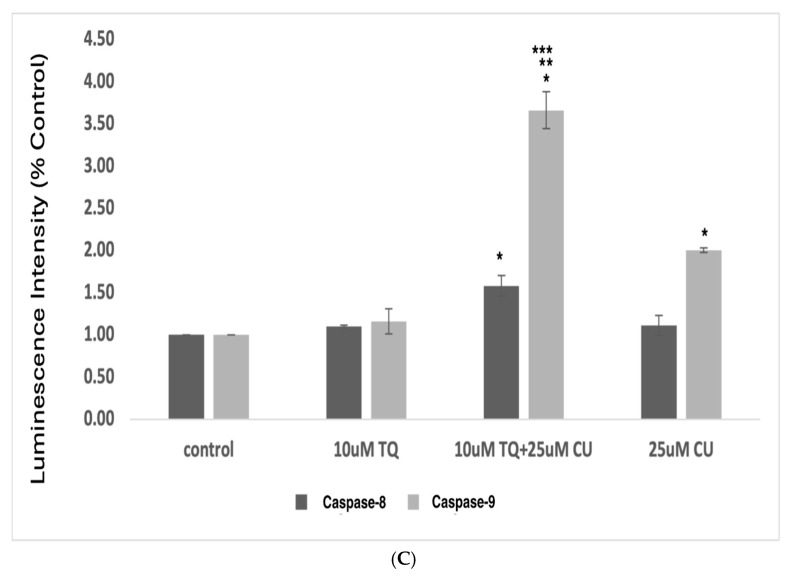
(**A**) Intracellular ROS levels in A375 cells after treatment with CU, TQ, and their combination. After the indicated treatment for 24 h, cells were incubated with 10 μM DCFH-DA for 30 min and then immediately subjected to a flow cytometry analysis. The results are expressed as a ratio of the relative fluorescence intensity compared to the control group. (**B**) The DCFH-DA spectrum represents the fluorescence intensities of the probe. DCFH-DA (10 μM) was incubated with the cell culture for 30 min. (**C**) Caspases-9 and -8 were measured using Caspase-Glo 8 Assay and Caspase-Glo 9 Assay kits. *p*-Values were recorded as the mean ± SD. * *p* < 0.05 vs. control cells; ** *p* < 0.05 combination vs. 25 μM CU; *** *p* < 0.05 combination vs. 10 μM TQ.

An increase in reactive oxygen species (ROS) is typical in mitochondria-related apoptosis [30]. Both the structural and functional integrity of mitochondria are crucial for maintaining redox balance [31]. TQ and CU significantly increase the production of ROS (Figure 5A,B). Caspase-8 is primarily associated with the extrinsic apoptotic pathway, started by binding death ligands (such as the Fas ligand or TNF-alpha) to death receptors on the cell surface. This binding leads to the formation of the death-inducing signaling complex (DISC), where caspase-8 is activated through proteolytic cleavage [32].

Caspase-9, conversely, is involved in the intrinsic apoptotic pathway. This pathway is initiated by intracellular signals, such as DNA damage or cellular stress, leading to the release of cytochrome c from mitochondria [33]. Cytochrome c then interacts with apoptotic protease activating factor 1 (Apaf-1) to form the apoptosome, which activates caspase-9 [34].

These data suggest that combining TQ and CU increases ROS production and caspase-9 activity more significantly than caspase-8 activity compared to individual treatments (Figure 5C). CU and TQ target different cellular pathways, leading to an overall increase in ROS production [35]. This was demonstrated in a study by Ullah et al., in which CU and TQ at low concentrations behaved as antioxidants, but at higher concentrations, they substantially inhibited the activity of essential antioxidant molecules responsible for scavenging free radicals [36]. Elevated levels of free radicals can result in DNA and protein damage [37].

Cancer cells exhibit elevated basal levels of reactive oxygen species (ROS), which promote tumor growth and progression [38]. However, excessive ROS levels can damage critical biomolecules, such as DNA and proteins, ultimately causing cell death. To survive, cancer cells rely on the increased expression of antioxidant enzymes [38]. Therefore, external sources of ROS or agents that induce oxidative stress may selectively target cancer cells [39]. For instance, the natural compound piperlongumine selectively induces cell death by targeting the oxidative pathway. A synergistic cytotoxic effect was observed when piperlongumine was combined with a pancratistatin analog, which activates the intrinsic apoptotic pathway through mitochondrial targeting [40].

The synergy between TQ and CU results in elevated ROS levels, preferentially activating the mitochondrial apoptotic pathway via caspase-9. Caspase-9 is a key player in the intrinsic apoptotic pathway, which is closely linked to mitochondrial integrity and ROS levels. CU has been shown to induce cell death in cervical and bone cancers through the activation of caspase-9 [41,42]. Similarly, TQ at higher concentrations induces cell death in human squamous carcinoma cells via caspase-9-dependent apoptosis [43].

These findings indicate that the combination induces cytotoxicity in melanoma cells driven by mitochondrial ROS generation and apoptosis induction through the mitochondrial pathway. Our findings were consistent with those of Soo et al. [44]. An increase in ROS is a typical phenomenon in mitochondria-related apoptosis. 

### 3.4. TQ and CU Regulate Differentially Expressed Genes (DEGs)

**Figure 6:**
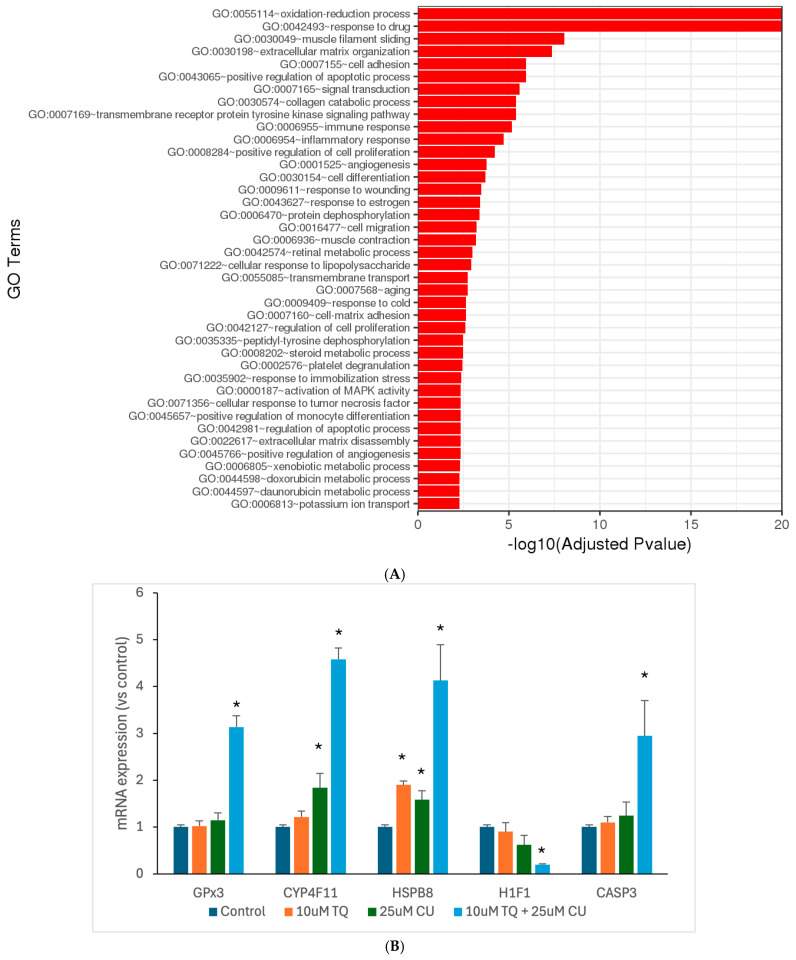
Significantly differentially expressed genes were clustered by their gene ontology, and the enrichment of gene ontology terms was tested using Fisher’s exact test (GeneSCF v1.1-p2): (**A**) significantly enriched gene ontology terms with adjusted *p*-values less than 0.05 in the differentially expressed gene sets (up to 40 terms) between 10 μM TQ+ 25 μM CU and the control; (**B**) mRNA expression of highly regulated genes, measured by RT-PCR and shown as the fold change compared to the control group. *p*-Values are presented as the mean ± SD. * *p* < 0.05 vs. control.

Motivated by the results of our in vitro assays, we aimed to investigate the underlying molecular mechanisms through which TQ and CU work synergistically against melanoma. Thus, we used RNA sequencing to look at the genome-wide changes in gene expression induced by either TQ, CU, or their combination in A375 cell lines. The DESeq2 of the total significantly differentially expressed genes relative to the untreated cells revealed 552 genes were commonly regulated by the combination treatment, 68 genes were uniquely regulated by CU, and 2 genes were uniquely regulated by TQ (Table 1). Gene ontology (GO) analysis is a widely used and effective approach for annotating genes and their products, as well as for identifying the biological features of high-throughput genome or transcriptome data [45]. TQ significantly negatively regulates blood vessel endothelial cell proliferation; this suggests that TQ may inhibit the growth of blood vessels that supply tumors, a process known as angiogenesis [46]. In addition, it is involved in the regulation of the apoptotic process, and this reinforces the idea that TQ may promote apoptosis, which is beneficial for melanoma treatment [15]. CU primarily affects processes such as the positive regulation of apoptotic, responses to drugs, and negative regulation of transcription from RNA polymerase II promoter, effectively preventing the initiation of gene expression [47] (Figure 6B). The combination treatment demonstrates a broad range of enriched GO terms related to oxidation-reduction processes, responses to drugs, apoptotic processes, and cellular responses to chemical stress, with a powerful impact on the oxidation-reduction process, indicating a significant effect on cellular redox homeostasis (Figure 6). 

The combination treatment’s marked enrichment of oxidative stress and redox processes suggests a more potent induction of oxidative stress, leading to increased ROS production. This aligns with the previous finding of higher ROS levels. Both TQ and CU individually influence apoptotic pathways. Still, the combination treatment exhibits a broader and more significant enrichment of apoptosis-related terms, indicating a more effective induction of apoptosis through multiple pathways. The combination treatment also exerts a more substantial effect on cell-cycle regulation, inhibiting cell proliferation more effectively than individual treatments, contributing to the synergistic effect of inducing cell death. Furthermore, the combination treatment enriches GO terms related to stress responses, such as responses to drugs and response to oxidative stress, indicating a heightened cellular stress response that could increase cancer cell susceptibility to apoptosis.

**Figure 7:**
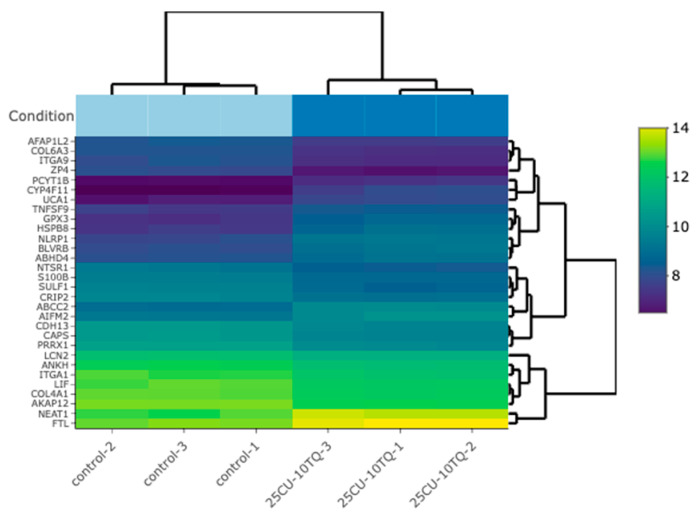
A bi-clustering heatmap for the combination vs. the control, which visualizes the expression profiles of the top 30 differentially expressed genes sorted by their adjusted *p*-values by plotting their log2-transformed expression values in the samples.

The investigation of the synergistic effects of TQ and CU on gene expression profiles in A375 cell lines reveals significant insights into their molecular mechanisms of action. The heatmap (Figure 7) analysis of the top 30 differentially expressed genes (DEGs) across the control, TQ, CU, and the combination treatments indicates distinct clusters for each treatment group, highlighting unique expression profiles. Notably, the combination treatment of TQ and CU forms a particularly distinct cluster, suggesting a unique synergistic effect on gene regulation, which has been corroborated by Korff et al. in previous studies on neuroblastoma cell lines, demonstrating that the combination of these compounds can enhance their anti-cancer efficacy through various signaling pathways [48]. The gene ontology (GO) analysis further supports this observation, indicating significant enrichment of processes related to oxidative stress and apoptosis, particularly with the combination treatment [49].

Table 1 shows the differentially expressed genes (DEGs) for TQ, CU, and their combination (Combo) treatments, which can be connected to the heatmap and gene ontology (GO) analysis to provide a comprehensive understanding of the molecular mechanisms underlying these treatments.

Only two genes, NGFR and ZP4, were significantly downregulated by the TQ treatment alone vs. the control. The downregulation of NGFR is involved in inhibiting cell viability and cell migration, aligning with findings that TQ can inhibit cell proliferation and migration in various cancer models [50]. ZP4, which is linked to cell proliferation, has been reported in colon cancer [51]. ZP4 also appears to play a role in immune modulation. Gorreja et al. confirmed that ZP4 is regulated by Toll-like receptors and contributes to the immune response, particularly in inflammatory contexts [52]. Knockdown studies revealed that silencing ZP4 altered the expression of pro-inflammatory cytokines, suggesting that ZP4 may influence the tumor microenvironment by modulating immune cell behavior [52]. The nerve growth factor receptor (NGFR) is a multifunctional cell surface receptor involved in diverse processes, including promoting cell survival and differentiation during neuronal development. Interestingly, NGFR has been implicated in tumor progression in certain cancers, such as brain tumors and melanomas. Its downregulation in response to TQ treatment is, therefore, significant, as it may help inhibit tumor progression and improve the therapeutic response [53]. This limited set of DEGs suggests that TQ alone has a focused impact, which aligns with the heatmap showing less pronounced gene expression changes compared to the combination treatment. The CU treatment shows a more extensive set of DEGs, with genes like ZP4 and ZFP36 being downregulated and overexpressed, respectively. These genes are involved in various processes, such as cellular stress response, transcription regulation, and reduces cell migration [54]. The downregulation of genes such as MMP9 leads to the inhibition of metastasis and migration [55]. The broader impact of CU on gene expression is reflected in the heatmap, with the CU-treated samples exhibiting more significant expression changes than the TQ-treated samples. 

The combination treatment significantly alters a comprehensive range of DEGs, including CYP4F11, ZP4, HSPB8, NLRP1, and GPX3, indicating a more comprehensive effect on cellular processes. Genes like CYP4F11 and GPX3 are involved in the oxidative stress response, supporting the GO analysis results that showed significant enrichment of the oxidative stress and redox processes for the combination treatment [56]. The heatmap corroborates this, with the Combo-treated samples displaying the most distinct and widespread gene expression changes. In the Combo treatment, upregulated genes, such as GPX3, CYP4F11, and HSPB8, are associated with the oxidative stress response, drug metabolism, and protein folding, respectively. This indicates an enhanced cellular response to stress and damage repair mechanisms. Additionally, downregulated genes like ZP4, TGM2, and MMP9 are primarily involved in the cellular structure and migration [57,58]. The downregulation of these genes suggests a reduction in cellular motility and invasiveness, which aligns with the GO terms related to cell proliferation and migration being impacted by the combination treatment.

The integration of DEGs with the heatmap and GO analyses provides a comprehensive view of how TQ, CU, and their combination impact A375 cell lines. The individual treatments, TQ and CU, have focused yet distinct effects on gene expression, with CU having a broader impact. The combination treatment, however, exhibits a synergistic effect, significantly altering a wide range of genes involved in oxidative stress response, apoptosis, and cell cycle regulation. This synergy is reflected in the GO enrichment analysis and visually represented in the heatmap, highlighting the enhanced efficacy of the combination treatment in targeting multiple cellular pathways for a more effective anti-cancer response. 

To validate the RNAseq data, we performed qPCR analyses for the GPX3, CYP4F11, CASP-3, H1F1, and HSPB8 genes (Figure 6B). The combination treatment significantly overexpressed GPX3, CYP4F11, CASP-3, and HSPB8, corroborating the RNAseq findings and the caspase assay. Wang et al. reported that the induced expression of GPX3 leads to increased ROS production and caspase-3 activity, thereby promoting apoptosis [59]. The anti-tumor effects of GPX3 were further demonstrated through its inhibition of HIF-1α and HIF-2α in melanoma cells, resulting from increased ROS scavenging by GPX3 [60]. The overexpression of HSPB8 can induce apoptosis in melanoma cells by activating transforming growth factor β-activated kinase 1 (TAK1) [61]. Notably, mutations in HSPB8 are associated with mitochondrial dysfunction and oxidative stress. According to findings by Yang et al., HSPB8 induces mitochondrial aggregation and exacerbates oxidative stress injury [62]. These findings suggest that the combination treatment acts as a pro-oxidative therapy, leveraging elevated oxidative stress to induce cell death in melanoma cells while disrupting their antioxidant defenses.

### 3.5. TQ and CU Drive Cellular Metabolomic Rewiring

**Figure 8:**
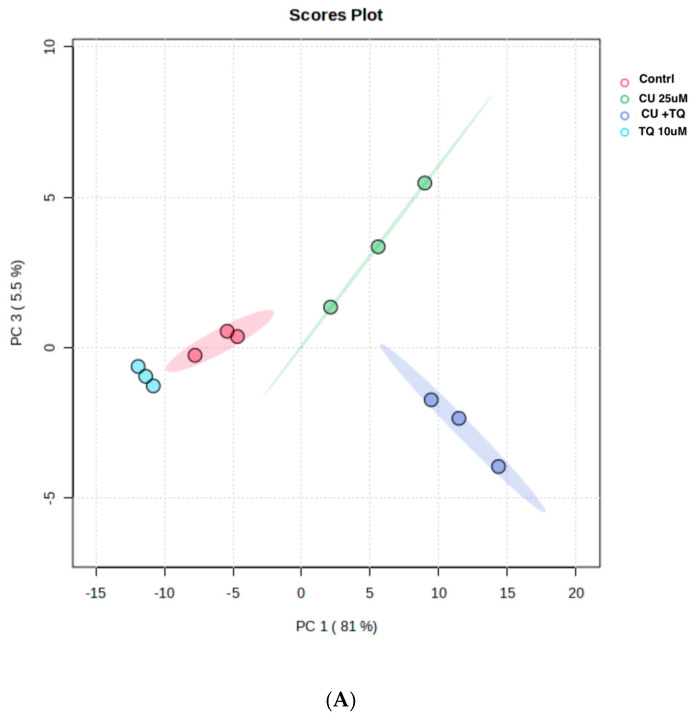

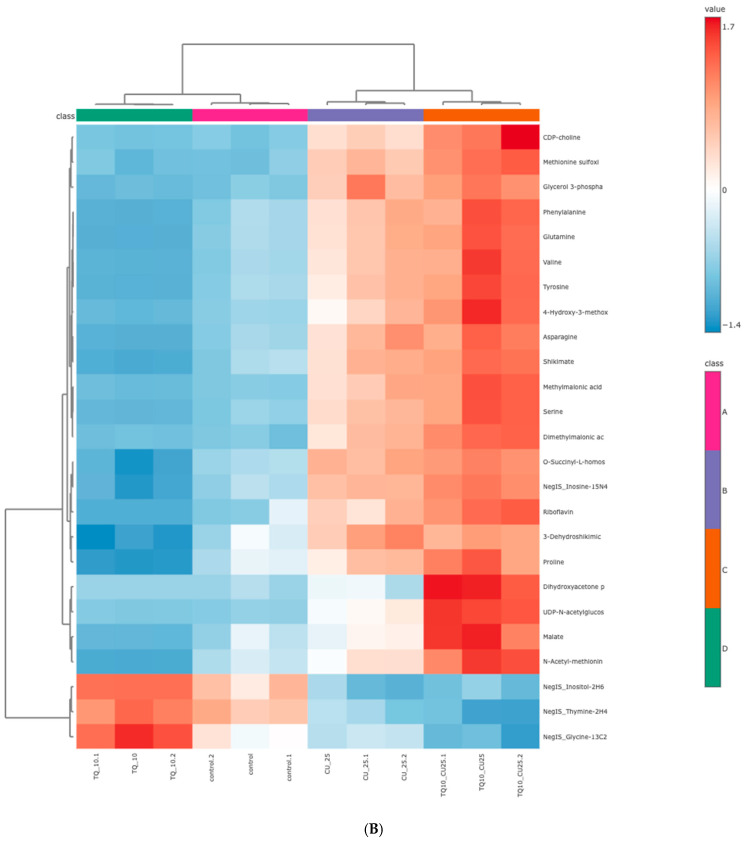

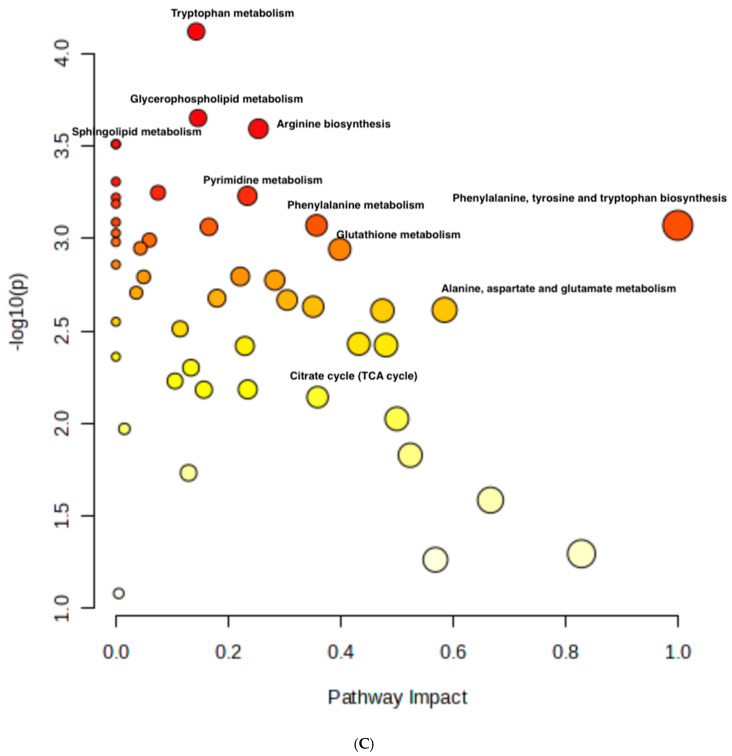
Metabolomics of TQ, CU, and their combination on treated melanoma cell lines: (**A**) PCA analysis of three groups showing that samples in each group are clustered away from one another; (**B**) heatmap of pairwise correlation values of 120 metabolites and depiction of the major metabolic pathways in A375 cells; (**C**) identified pathways altered by the combination treatment vs. the control treatment in A375 cell lines.

The RNAseq data and metabolomic pathway analysis provide complementary insights into the effects of thymoquinone (TQ) and curcumin (CU) on A375 cell lines, revealing a comprehensive mechanism of action against melanoma cells. Metabolic reprogramming, a hallmark of tumors, plays a crucial role in tumorigenesis and signaling and in anti-tumor effects through the release of metabolism-related factors such as lactate, arginine, and tryptophan. An untargeted metabolomics approach using LC-MS was employed to investigate the metabolic profile of A375 cells treated with TQ, CU, and their combination. The principal component analysis (PCA) performed on all samples showed that the groups were tightly clustered in the PCA score plot, indicating clear distinctions among the treatments (Figure stheir combination had strong effects, resulting in significant metabolic variations in A375 cells (Figure 8B). The heatmap analysis of upregulated and downregulated genes, alongside the gene ontology enrichment and metabolomic pathway analysis, provides a comprehensive understanding of the mechanism of action of thymoquinone and curcumin in killing melanoma A375 cell lines. The heatmap showed that upregulation of metabolites in the treated groups, such as riboflavin, methionine, and glycerol-3-phosphate, led to increased lipid peroxidation due to cell apoptosis.

These are involved in apoptosis and oxidative stress response, indicating the treatment’s role in disrupting cellular homeostasis in melanoma cells. CU has been reported to disrupt glutamine metabolism, reducing tumor growth availability, and promoting apoptosis in melanoma cells [63]. The alteration of several metabolic pathways has been shown to induce mitochondrial dysfunction in cancer cells, leading to decreased ATP production and increased oxidative stress, which can trigger apoptosis [64]. CU’s and TQ’s ability to enhance oxidative stress in cancer cells has been well documented, and this may lead to increased riboflavin levels as a compensatory mechanism [15,65].

The metabolomic analysis further highlighted several key pathways impacted by the treatments, such as the tryptophan metabolism, glycerophospholipid metabolism, sphingolipid metabolism, and arginine biosynthesis. Other affected pathways included pyrimidine metabolism, phenylalanine metabolism, glutathione metabolism, citrate (TCA cycle), alanine, aspartate, and glutamate metabolism (Figure 8C). These pathways are crucial for cell survival, proliferation, and energy production, and their disruption aligns with the gene expression changes observed.

For instance, glycerophospholipid metabolism is recognized as a critical factor in cancer progression, with alterations in this pathway being linked to tumor migration and metastasis [66,67,68]. Moreover, the connection between tryptophan metabolism and apoptosis has been highlighted, suggesting that the combination treatment may induce oxidative stress and promote apoptotic processes in cancer cells [69]. 

The integration of RNA sequencing and metabolomic data reveals that the treatment primarily kills cancer cells by inducing oxidative stress, enhancing apoptotic processes, and disrupting critical metabolic pathways. Several pathways are connected to gene regulations after the combination treatment, such as PCYT1B, ABHD4, CYP4F11, and GPX3, which are connected to tryptophan metabolism, sphingolipid metabolism, arginine biosynthesis, and glutathione metabolism, respectively, indicating a direct link between metabolic alterations and gene regulation following treatment.

The RNAseq data and metabolomic analysis indicated changes in the redox states, which can lead to oxidative stress and trigger apoptosis in cancer cells. The GO analysis showed enhanced apoptosis, while the metabolomic data revealed disruptions in tryptophan metabolism, glycerophospholipid metabolism, and sphingolipid metabolism, which are vital for cellular energy production and biosynthesis.

## 4. Conclusions 

The combination of CU and TQ synergistically inhibits cell viability, induces apoptosis, and disrupts metabolic and oxidative homeostasis in melanoma cells. Our study highlights the significant role of mitochondrial ROS generation and apoptotic signaling in mediating the anti-tumor effects of the combination. Moreover, the upregulation of key oxidative-stress- and apoptosis-related genes, such as GPX3, CYP4F11, and HSPB8, provides insight into the molecular mechanisms underlying this combination’s effectiveness. While these findings strongly support the potential of CU and TQ as a combined therapeutic strategy, further studies are needed to elucidate the precise mechanism of action and molecular pathway involved and to validate these results in preclinical and clinical models. This future work will pave the way for optimizing the use of this combination in melanoma management.

## Figures and Tables

**Table 1:** Summary of the differentially expressed genes among the groups, analyzed using DESeq2. The Wald test was applied to obtain the *p*-values and log2 fold changes. Genes with an adjusted *p*-value of less than 0.05 and an absolute log2 fold change of greater than 1 were identified as differentially expressed.

Comparison	Upregulated Genes	Downregulated Genes
TQ vs. Control	0	2
TQ vs. CU	50	15
TQ vs. (TQ + CU)	284	114
CU vs. Control	37	31
CU vs. (TQ + CU)	211	74
(TQ + CU) vs. Control	269	283

## Data Availability

The data presented in this study are available on request from the corresponding author due to due to privacy/ethical restrictions or institutional policies.

## Abbreviations

Thymoquinone (TQ); curcumin (CU); Combination Index (CI); reactive oxygen species (ROS); differentially expressed genes (DEGs); gene ontology (GO).
